# Gastrointestinal nervous system α-synuclein as a potential biomarker of Parkinson disease

**DOI:** 10.1097/MD.0000000000011337

**Published:** 2018-07-13

**Authors:** Fudong Yan, Ying Chen, Min Li, Yingqing Wang, Wenmin Zhang, Xiaochun Chen, Qinyong Ye

**Affiliations:** a Department of Neurology, Fujian Institute of Geriatrics, Fujian Medical University Union Hospital; b Hospital of Fujian Shunchang; c Department of Pathology, School of Basic Medical Sciences; d Key Laboratory of Brain Aging and Neurodegenerative Diseases, Fujian Key Laboratory of Molecular Neurology, Fujian Medical University, Fuzhou, China.

**Keywords:** α-synuclein, gastrointestinal nervous system, Parkinson disease

## Abstract

Lewy bodies (LB) play an essential role in the development, survival, and function maintenance of midbrain dopaminergic (DA) neurons in Parkinson disease (PD). Alpha-synuclein (α-synuclein) is the major component of Lewy bodies and is a potential target for Parkinson's disease (PD) therapies. α-synuclein can be detected in the gastrointestinal (GI) nervous system, but whether there is any association between altered α-synuclein expression in the GI nervous system and the onset of PD is not known. The answer to this question presents the opportunity for a promising biomarker in the pre-clinical diagnosis of PD. As such, this study aimed to measure the α-synuclein level in the GI nervous system of Parkinson's disease patients.

The protein levels of α-synuclein in the GI nervous system of 31 PD patients (PD group) and 32 patients without PD or Parkinsonism-plus syndrome (control group) were evaluated via immunohistochemical staining. The *χ*^*2*^ test was performed to evaluate the differences between the PD group and control group. In addition to the distribution of α-synuclein positive protein, regional distribution of the protein in the stomach was also evaluated across groups.

Alpha synuclein overexpression was found in the GI nervous tissue of PD patients. The PD group included 17 positive results and 14 negative results. The control group exhibited 7 positive results and 24 negative results. The *χ*^*2*^ test showed that *χ*^*2*^ = 7.255*, P* = .01. The distribution of these positive cases in the gastrointestinal system, the *χ*^*2*^ test showed that *P* = .949. The 21 stomach tissues had 7 α-synuclein positive protein tissues, while the body of stomach (4 α-synuclein positive protein) was higher than in other regions.

Aberrant expression of α-synuclein was detected in the GI tissues of PD patients, though the distribution of α-synuclein in the gastrointestinal tract had no specificity. Gastrointestinal mucous biopsy could be regarded as a potential opportunity for the early-stage diagnostic exploration of PD, through the detection of α-synuclein inclusions.

## 1 Introduction

Parkinson disease (PD) is the second most common progressive degenerative disease of the nervous system in middle-aged and elderly people. The etiology and pathogenesis of PD are still not clear. Currently, it is supposed that during aging, complex interactions between environmental and genetic factors lead to multiple mechanisms including misfolding, abnormal aggregation, and corresponding abnormal degradation of proteins. These events in turn lead to protein aggregation, immune-inflammatory reaction, oxidative stress, mitochondrial dysfunction or defect, calcium overload and apoptosis, culminating in massive degeneration, and loss of nigral dopaminergic neurons.^[1,2]^

Clinically, PD is characterized by pathological changes such as the degeneration and loss of dopaminergic neurons in the midbrain substantia nigra, and gliosis combined with the formation of Lewy bodies (LB), which are the gold standard for PD diagnosis.^[3]^ LBs are mainly constituted by α-synuclein,^[4]^ a protein which consists of 140 amino acids composed of the carboxyl terminal (96–140), the NAC region (6l–95), and the amino terminal (1–60) that plays a particular role in the aggregation of α-synuclein. The α-synuclein, a natural, non-folding, and soluble protein with a molecular weight of 14 kD, is mainly distributed in neurons and highly expressed in the presynaptic terminals of the central nervous system.^[5,6]^ Moreover, α-synuclein is widely expressed in nervous tissue, with a higher content in the olfactory bulb, hippocampus, neocortex, cerebral ganglia, and corpus striatum, while a lower content in the brainstem.^[7,8]^ Increasing evidence indicates that PD is also involved in the dopamine system of other organs (such as pathways in the mesolimbic system, mesocortical pathways, and dopaminergic neurons in the gastrointestinal tract), the serotonergic system, cholinergic system, noradrenergic system, and peptide neurotransmitter systems (such as enkephalin system and dynorphin system), likely underlying a series of non-motor PD symptoms.^[9–12]^

The enteric nervous system is a complete neural network which exists in the gastrointestinal wall and is independent of the brain.^[13–15]^ It is composed of a large number of neural cells whose processes are buried in the gastrointestinal wall.^[13–15]^ Neural cells cluster, and the enteric ganglia forms 2 main ganglionated plexuses through the connection of the nerve cell processes, namely myenteric nerve plexus and submucosal nerve plexus.^[13–15]^ Minguez-Castellanos et al^[16]^ carried out anti- alpha-synuclein immunohistochemical staining on pathological specimens from 100 patients (44–84 years old) after the wide excision of abdominal and pelvic organs in 2007. From a cohort of these patients, it was revealed that the aggregation of α-synuclein in peripheral autonomic neurons occurs earlier than the development of LB disorder.

The cellular hallmarks of PD include the loss of dopamine neurons in the substantia nigra, the formation of LB in α-synuclein aggregation.^[17]^ After establishing a theory based on the local anatomical distribution of LB through the autopsy of the brain in patients with PD, Braak et al^[12]^ assumed that the early formation of LBs occurred in the gastrointestinal tract, gradually spreading to the brain, which suggested the presence of jumping retrograde axoplasmic transport of α-synuclein through vagus. In order to confirm this hypothesis, Holmqvist et al^[18]^ implanted the brain lysates of PD patients, including α-synuclein in different forms (monomer, oligomer, and fibrous) and recombinant α-synuclein, into an in vivo animal model. This experiment evidenced that after the injection of α-synuclein into the wall of the small intestine, α-synuclein could arrive at the dorsal nucleus of the vagus nerve in the brainstem, a phenomenon which exhibited a time-dependence.^[18]^ Moreover, live cell imaging in a differentiated neuroblastoma cell line demonstrated the coexistence of 2 axonal transmissions (rapid and slow) that were used in the transport of aggregated α-synuclein. Additionally, the prion-like hypothesis of α-synuclein progression for the pathogenesis of PD has proposed α-synuclein that can be transferred between cells, rather than LBs through prion mechanisms.^[18]^ Evidence revealing that PD was avoided in all patients who received complete vagal denervation similarly supports the notion of α-synuclein transport.^[19]^

In the pathology of PD, the abnormal deposition of α-synuclein mainly appears in the central nervous system; however, it is noted recently that the aggregation of α-synuclein can be detected in the enteric nervous system by colonoscopy biopsy.^[20,21]^ In 2012, researchers found that before the occurrence of symptoms in the patients with advanced PD, colon biopsy had been able to find α-synuclein, demonstrating that α-synuclein protein in the intestinal wall may be an early marker for PD.^[22]^ Sánchez-Ferro et al^[23]^ also detected the aggregation of α-synuclein in the stomatogastric nervous system of PD patients by gastroscopy biopsy in 2014. In fact, Shannon KM et al^[22,24]^ proposed that the enteric nervous system might be the earliest involved structure in the neurodegenerative process in PD, which is different from the previous notion that degradation initiates from the central nervous system.

### 1.1 Objectives

The present study was designed to detect the expression of α-synuclein through the peripheral nerves of the gastrointestinal tract and nerve plexuses in patients with PD, to verify the abnormal aggregation of α-synuclein in the peripheral nervous system, providing a biological marker for the early diagnosis of PD in the future.

## 2 Methods

### 2.1 Selection of subjects

Thirty-one patients were recruited to the PD group from a clinical population of patients with sporadic PD and gastrointestinal pathological tissue who visited the Inpatient Department and the Outpatient Department of the Union Hospital Affiliated to Fujian Medical University between March 2007 and March 2014.

A diagnosis of PD was ascribed when patients met the diagnostic criteria for primary PD formulated by the Brain Bank of the British Association of PD,^[3]^ and the diagnostic criteria for PD formulated by the Movement Disorders and Parkinson Disease Group of the Neurology Branch of Chinese Medical Association.^[25]^ PD was diagnosed by 2 attending physicians or physicians in a higher position of the Department of Neurology. A PD diagnosis constituted the inclusion criteria. The exclusion criteria included patients who exhibited a failure in levodopa treatment, secondary Parkinson syndrome, Parkinsonism-plus syndrome, or other diseases that may cause dyskinesia. Additionally, patients who did not complete the experimental process were excluded from the study.

Pathological sections were collected from tissue of the digestive tract (stomach, intestine, and appendix).

**Figure 1:**
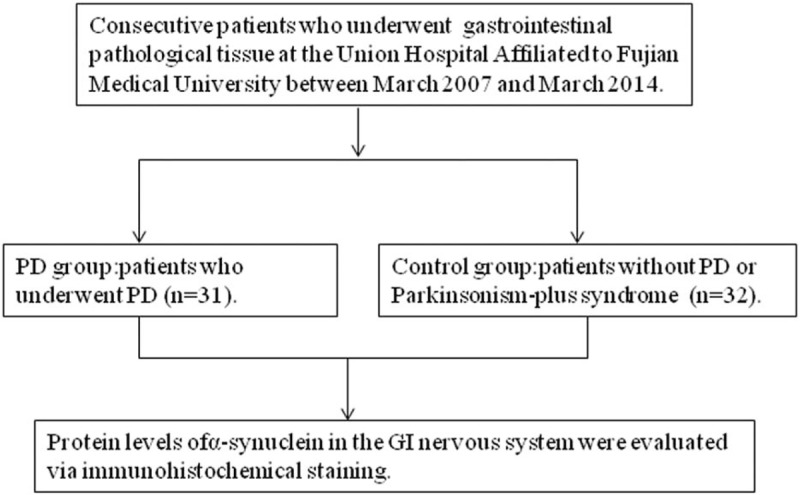
Flow chart illustrating selection of the study groups.

Thirty-two patients without PD or Parkinsonism-plus syndrome who visited the Inpatient Department and the Outpatient Department of the Union Hospital Affiliated with Fujian Medical University between March 2007 and March 2014 were additionally recruited into the control group. The 2 groups were matched in gender, age, diseases, and location of pathological resection (Table 1). Flow chart illustrating selection of the study group is presented in Figure 1.

**Table 1:**
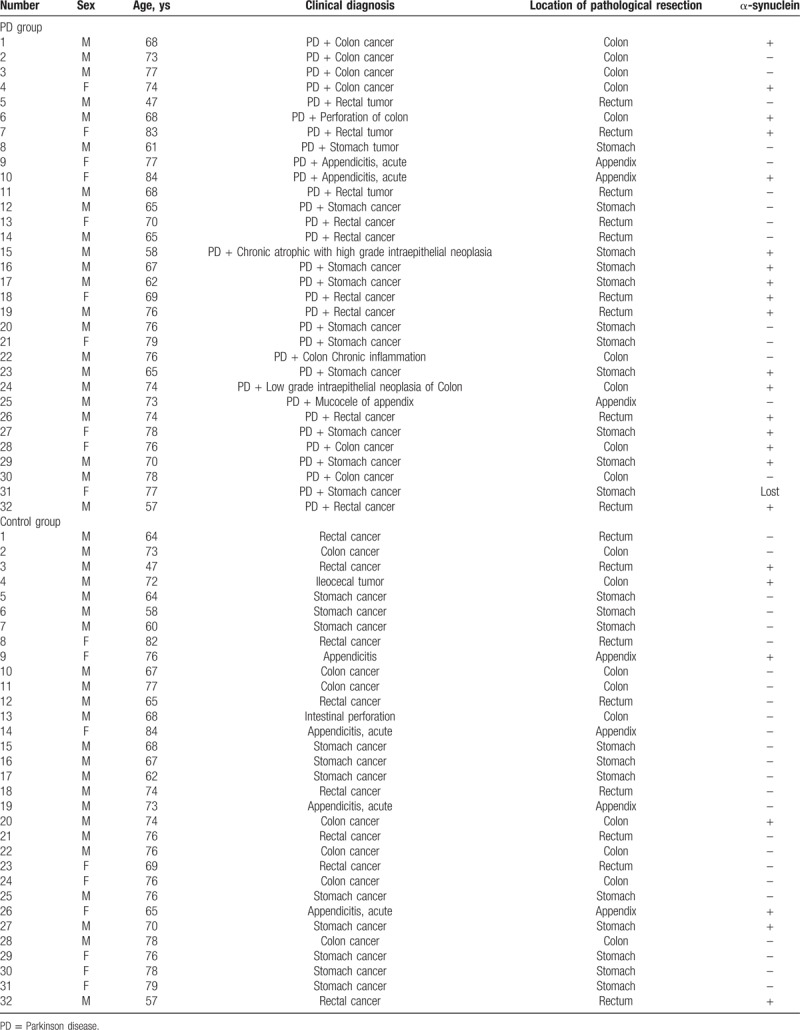
Demographic, clinical, and neuropathological data from PD group and control group.

This study was approved by the Ethics Committee of the Union Hospital Affiliated to Fujian Medical University, and all subjects or their family members signed informed consent prior to study enrollment.

### 2.2 Clinical evaluation

Patient survey and registration of general information included age, gender, smoking history, age of onset, initial symptoms, family medical history, time of onset, history of alcohol use, disease severity at that time of evaluation, drug therapy, non-motor symptoms, and other accompanied diseases (which were further confirmed when signing informed consent). Evaluation of motor function of all PD patients was evaluated using Part 3 of the Unified Parkinson Disease Rating Scale (UPDRS).

### 2.3 Experimental methods

#### 2.3.1 Instruments and reagents

Main instruments used in this study included a Leica RM2245 microtome (Leica Microsystems GmbH, Wetzlar, Germany) and Constant temperature oven maintained at 65°C (Shanghai Instrument Manufacturing Factory, Shanghai, China). A BX53 optical microscope (Olympus, Tokyo, Japan) was used in conjunction with Beion software analysis system for medical imaging (Shanghai Beion Medical Technology Co. Ltd., Shanghai, China).

##### 2.3.1.1 Reagents

Reagents for study protocols included α-synuclein mouse monoclonal antibody (Santa Cruz Biotechnology, Santa Cruz, California), Elivision TM plus secondary antibody (KIT 9902) (Fuzhou Maixin Biotechnology Development Co., Ltd, Fuzhou, China), Citric acid antigen repair solution (concentrate MVS-0066) (Fuzhou Maixin Biotechnology Development Co. Ltd.), DAB package (Fuzhou Maixin Biotechnology Development Co. Ltd.), Immunohistochemical pen (No.: PEN-0002) (Fuzhou Maixin Biotechnology Development Co., Ltd.), and normal non-immune rabbit serum (SP KIT-B2) (China ELISA kit sales platform). Additional reagents included xylene (Lanzhou Longxi petrochemical Co., Ltd., China), 100% alcohol (Lanzhou Longxi petrochemical Co. Ltd., Lanzhou, Gansu, China), hydrogen peroxide (Lanzhou Longxi petrochemical Co., Ltd.), and 4% paraformaldehyde.

#### 2.3.2 Immunohistochemistry

Pathological tissues were collected from the paraffin-embedded stomachor intestine or appendix specimens, respectively, from 31 PD patients and 32 controls. Prior to immunohistochemical evaluation, each paraffin block was sectioned serially into serial slices (thickness, 3.5 μm), and 2 sections were selected and mounted onto anti-slip slides and baked in a, 65°C oven for 90 minutes. Next, dewaxing of sections was performed according to the following incubations: xylene 10 minutes → xylene 10 minutes → 100% alcohol 5 minutes → 100% alcohol 5 minutes → 95% alcohol 5 minutes → 80% alcohol 5 minutes → 75% alcohol 5 minutes. After the hydration with gradient alcohol, the sections were washed with PBS 3 times for 3 minutes each time. Tissue sections were incubated in 50 μL 3% hydrogen peroxide solution at room temperature for 10 minutes to block endogenous peroxidase activity. Next, citric acid was used for high-temperature, high-pressure antigen retrieval according to the following: citric acid antigen repair solution (1000 mL) was poured into a pressure cooker, which was placed on a high power (2000 W) electromagnetic oven until solution came to a boil.

Sections were placed on a high temperature-resistant plastic staining rack and placed into the baked solution, with lid covered and pressure valve buckled, followed by continuous heating. When the pressure valve began to change, 2 minutes were allowed to elapse and the pressure cooker was removed from the electromagnetic oven and left to stand at room temperature for 10 minutes. When the pressure in the pressure cooker returned to the atmospheric pressure, the pressure valve was removed, and the pressure cooker was cooled by running water. After solution cooled to room temperature, the sections were taken out and washed with PBS. After drying, immunohistochemistry pen was used to draw circles at 3 mm from the tissue.

Each section was incubated with 50 μL 10% normal non-immune rabbit serum at 37°C for 10 minutes. Next, sections were incubated in 50 μL α-synuclein primary antibody (1: 100) at 4°C overnight. The following day, sections were washed 3 times with PBS for 3 minutes and subsequently incubated in 50 μL regent A (polymer reinforcing agent) at room temperature for 20 minutes. After PBS wash, 50 μL of reagent B (enzyme-labeled anti-mouse/rabbit polymer) was added and sections incubated at room temperature for 30 minutes. After PBS wash, 50 μL fresh DAB were added and sections were observed under the optical microscope for 6 minutes.

After final washing with distilled water, the sections were counterstained with haematoxylin, differentiated with 0.1% hydrochloric acid when necessary, returned to blue with PBS, and immersed in distilled water for 3 minutes. After drying and dehydration with gradient alcohol, the pathological sections were preserved with xylene and then sealed with neutral gum.

#### 2.3.3 Evaluation of α-synuclein

Under the light microscope, comparison of positive and negative sections were performed in a double-blind method. The determination of experimental results was carried out to clarify whether the sections were positive, negative, or nonspecifically stained. Previously, researchers reported that the submucosal layer was the site where α-synuclein aggregation was the most easily found.^[24]^ As such, nerves and nerve plexuses were our target sites in the mucosa, submucosa and muscle, with brown-stained cytoplasm indicative of positive cells and non-brown-stained cytoplasm as negative cells. Positive and negative cells were counted under a high-power field (10 × 40) and scored according to intensity firstly: non-stained, 0; pale-yellow fine particles, 1; pale-brown particles, 2; and brown yellow coarse particles, 3. Next, the staining was scored based on the percentage of positive cells: no positive cells, 0; the percentage of positive cells < 10%, 1; the percentage of positive cells between 10% and 20%, 2; the percentage of positive cells between 20% and 50%, 3; the percentage of positive cells > 50%, 4. The sum of the score of staining intensity and positive cell rate was combined to determine the score of the patient. Overall, a patient with a score of 0 to 2 was considered as negative, and a patient with a score equal or greater than 3 was considered positive.^[26]^

### 2.4 Statistical methods

All data were expressed as mean ± standard deviation (X ± S). Comparison in each group and between groups was conducted by analysis of variance and the q test; ranked data were compared by the rank-sum test. Comparison between 2 groups was carried out by *χ*^*2*^ test, and *P* < .05 was considered as statistically significant. SPSS 20 statistical software (International Business Machines, Armonk, NY) was used for comparison and analysis.

## 3 Results

### 3.1 General clinical information

All 64 patients (1 pathological tissue of a female patient in the PD group was lost during dewaxing and antigen repair) underwent surgical resection, with 31 patients in the PD group (23 males and 8 females) and 32 patients in the control group (23 males and 9 females). The age of all patients ranged from 47 to 84 years old, with an average age of 70.6 ± 8.1 years old in the PD group and 70.3 ± 8.1 years old in the control group. There was no significant difference in age between both groups (*P* = .908). In the PD group, the duration of the disease was 0.5 to 15 years, with the longest duration in a 74-year-old male patient. Regarding clinical manifestations, tremor was identified in 21 patients, rigidity in 15 patients, slow walking in 13 patients, and abnormal posture, and gait in 11 patients, the above symptoms overlapped each other. Among the 31 patients in the PD group, 2 had acute appendicitis, 1 had appendiceal mucocele, 1 had chronic atrophic gastritis accompanied by high-grade intraepithelial neoplasia, 1 had colonic perforation, 1 had transversostomy chronic inflammation, 9 had gastric cancer, 7 had colon cancer, and 9 had rectal cancer. Additionally, there were 11 patients with non-cancer and 5 with appendicitis. There were no statistical differences in age, gender, sampling site, or diseases between the PD group and the control group.

### 3.2 Immunohistochemical results of α-synuclein

There were a total of 126 sections included for immunohistochemical evaluation of α-synuclein. Two pathologists performed the observation by a double-blind method, revealing that sepia aggregate (namely α-synuclein aggregate, positive) was mainly found in the submucosal layer, and intramuscular nerves and nerve plexuses in 24 pathological tissues. On the other hand, no sepia aggregate (namely no α-synuclein aggregate, negative) was found in 39 pathological tissues. (Immunohistochemical staining was conducted in at least 2 sections for each patient to avoid false negatives during the experimental process.α-synuclein aggregates presented in one or more of the sections, which was considered as a positive result. According to these parameters, the PD group showed 17 positive results and 14 negative results while the control group showed 7 positive results and 25 negative results. The statistics were shown in Table 2. The *χ*^*2*^ test showed that *χ*^*2*^ = 7.255*, P* = .01 < .05, suggesting a significant difference, mainly that the expression of α-synuclein aggregate increased significantly in the PD group.

**Table 2:**
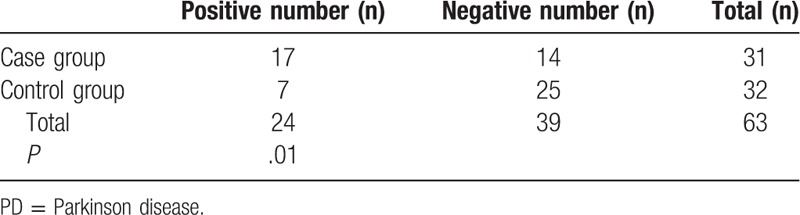
Positive expression of α-synuclein in nervous tissues of the gastrointestinal tract in PD group and control group.

**Figure 2:**
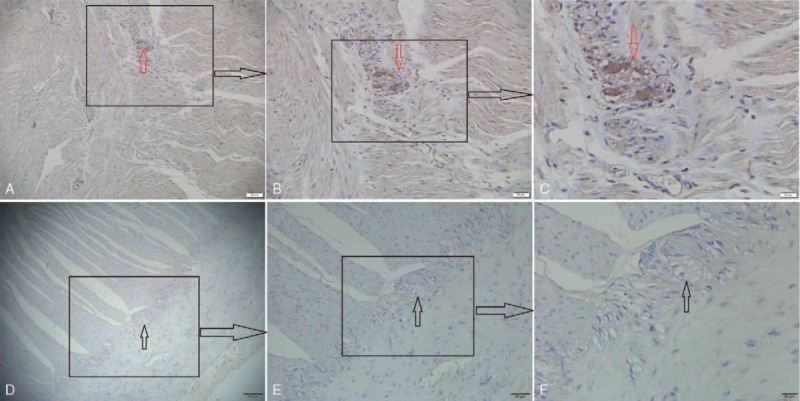
The α-synuclein immunoreactivity in sections of intestinal submucosal intramuscular nerve plexuses. Expression of α-synuclein in the intramuscular nerve plexus of the intestinal submucosa, PD group: ABC (positive: red arrow), control group: DEF (negative: black arrow) (A × 100, B × 200, C × 400, D × 40). ABC: Distinct α-synuclein expression increased markedly in the submucosal intramuscular nerve plexuses, as the immunoreactive materials of α-synuclein were granularly distributed in the intramuscular nerve plexuses, mainly in the cytoplasm of the intramuscular ganglions, though a few granular positive structures were also scattered in the nerve fibers. DEF: No distinct α-synuclein expression increased markedly in the submucosal intramuscular nerve plexuses.

Further, under the fields of × 100, 200, and 400, observation and photographing were conducted by randomly selecting 5 non-overlapping fields for each group. Representative observations can be seen in Figure 2, which showed the intestinal tissue of a 68-year-old male patient with PD for 6 years. Our observation revealed that α-synuclein expression increased markedly in the gastric and intestinal submucosal intramuscular nerve plexuses, as the immunoreactive materials of α-synuclein were granularly distributed in the intramuscular nerve plexuses, mainly in the cytoplasm of the intramuscular ganglions, though a few granular positive structures were also scattered in the nerve fibers. We can get the same observation in all the intestinal tissue of patient with PD or without PD.

In order to better understand the distribution of these positive cases in the gastrointestinal system, we carried out a statistical analysis of various locations of the positive and negative cases, as shown in Table 3. The χ^2^ test showed a *P* = .949, suggesting that the distribution of α-synuclein in the gastrointestinal tract had no specificity.

**Table 3:**
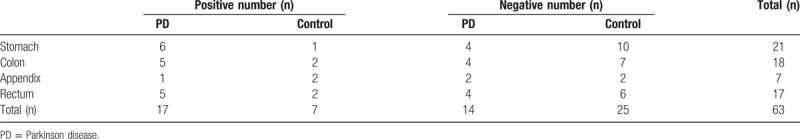
The distribution of α-synuclein positive protein of the gastrointestinal tract in PD group and control group.

On the other hand, some previous investigations have found that sections from different parts of the stomach exhibited differential sensitivity and specificity. In Table 4, however, we present the results of our statistical analysis of α-synuclein expression in different parts of the stomach. Our study revealed that α-synuclein expression in the body of the stomach was higher than in other regions.

**Table 4:**
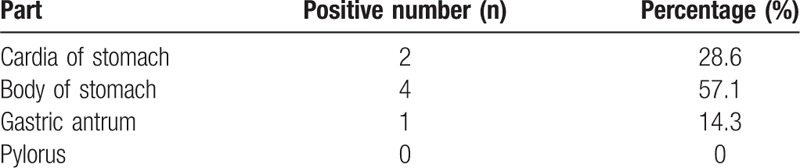
The expression of α-synuclein positive protein in different parts of the stomach.

## 4 Discussion

James Parkinson originally put forward that the pathology of PD originated in the brain.^[27]^ However, when patients are clinically diagnosed as PD, more than 50% of substantia nigra neurons in the brain have already been lost and there is limited neural protective intervention.^[28]^ Therefore, in the early stage of PD, a biological indicator that can be obtained conveniently from the peripheral tissue of PD patients and measured objectively would be valuable in preclinical phases to significantly optimize the diagnostic rate.^[29]^

Recent evidence has illustrated that the detection of α-synuclein aggregates in the enteric nervous system can serve as a potentially useful and novel diagnostic target for neurodegenerative diseases,^[22,23]^ and that α-synuclein aggregates also exist in the gastric nervous system of PD patients.^[24]^ The latest evidence also suggests that the pathology of PD may occur from the gastrointestinal tract to the brain, as demonstrated in a mouse model.^[18]^ Therefore, the detection of α-synuclein aggregates in the stomach and intestine may serve as a peripheral biological marker for PD diagnosis.

In this study we detected the expression of α-synuclein aggregates in the gastrointestinal nervous system of PD patients, and conducted a comparison of the protein between PD patients and control group. There were no statistical differences in age, gender, sampling site, pathological time or diseases between both groups. Our results revealed that α-synuclein aggregates could be detected in the gastrointestinal nervous system of PD patients at a higher rate than controls.

Among the 31 cases, 17 were classified as α- synuclein positive, accounting for 54.8%. These results, by comparison, are slightly lower than the positive rate of 60.7% previously reported by Sánchez-Ferro et al^[23]^ in the gastric nervous system of PD patients. Additionally, we suspect the false negative rate is relatively high, and may undermine the actual rate of α-synuclein aggregation due to the following: sampling error due to the scattered distribution of α-synuclein positive protein in the gastrointestinal system, pathological tissue specimen processing may result in a loss of α-synuclein protein, the late stage degeneration of PD may obscure positive detection, or obvious submucosal layer staining may not be detected in some pathological tissues under an optical microscope. Several reports have also demonstrated routine 4 to 6-μm-thick paraffin sections of the GI biopsies do not enable visualization and a minimum cutoff of 4 slides per case to reliably find ASN-IRS in PD patients in a study examining gastric biopsies.^[30]^

Among the 32 controls, it was detected that 7 samples showed positive immunoreactivity accounting for 21.9% of the sample, which was relatively high. There are several reasons which may underlie this false positive phenomena. For example, due to study recruitment methods, it could not be ruled out whether controls without clinical motor PD symptoms were simply patients in the early stages of PD without long-term patient follow-up. Along the same lines, there may exist a disease spectrum of synucleins, such as in multiple system atrophy, Dementia with Lewy bodies (DLBs), Alzheimer's disease, nerve degeneration with iron ion deposition (type N BIA 1), simple autonomic nervous breakdown, type I spinal cerebellar ataxia, subtype specific tremor, and so on,^[31]^ among whom positive results may have occurred. Moreover, previous research has shown α-synuclein aggregates in the brains of more than 10% of normal aging patients and recent studies appear to corroborate this idea, showing that this may be a marker of PD before the onset of symptoms.^[32]^

Another important finding in this study was the expression of α-synuclein aggregates in different parts of the gastrointestinal tissue, such as the stomach, appendix, colon and rectum. Mainly, we report no specificity of the expression of α-synuclein among different regions. Interestingly, we did find α-synuclein aggregates in the submucosa of the appendix in 1 patient in the PD group and 2 patients in the control group. These cases, though unique, do support similar findings by Gray et al,^[33]^ who also found α-synuclein aggregates in the appendix, in the mucosal nerve plexuses rather than classic submucosal or intestinal myenteric plexuses. Further, while α-synuclein was not differentially distributed in the gastrointestinal tissue, statistical assessment revealed that α-synuclein was preferentially distributed in the body of the stomach, which provides an important basis for the selection of the sampling sites of gastrointestinal tissues in the future.

As for the application of antibodies, there is currently no consensus on the usage of α-synuclein antibodies in PD. Croisier et al^[34]^ tested 7 commercial anti-α-synuclein monoclonal antibodies, showing that those amino acids in 116 to 131 and 15 to 123 seem to be the optimal, and recommended for routine use in the diagnosis of synuclein diseases. In our study, the selected antibodies with amino acids in 121 to 125 were within the recommended range, and demonstrated good sensitivity and specificity. However, some scholars believe that the 5G4 antibody is the most promising one.^[35]^ We did not carry out a control study based on these different antibodies, which may have had an influence on our immunohistochemical results.

Some other study limitations should be acknowledged. First, the cross-sectional case-control design of our study contributed to a small sample size. Additionally, in the control group, there were many gastrointestinal symptoms which were difficult to distinguish from the non-motor symptoms of PD. Finally, the site of pathological sampling could not be fixed; for instance, a certain site of the stomach or intestine could not always be isolated uniformly for sampling.

This study systematically investigated the expression of α-synuclein in the nervous system of the entire gastrointestinal tract (stomach, colon, rectum, and appendix) of PD patients, with a positive rate reaching 54.8%. Our findings support the notion that PD is actually a neurodegenerative disease involving a number of systems, and that pathological α-synuclein aggregates are not only limited to the brain, but also exist in the gastrointestinal nervous system. The α-synuclein aggregates in the gastrointestinal nervous system may in the future be used as a biological marker for the early diagnosis of PD. For those patients with constipation, abnormal sense of smell, rapid eye movement sleep behavior disorder or unclear diagnosis, the detection of α-synuclein aggregates in the gastrointestinal tract may contribute to the early diagnosis of PD, or provide help for the diagnosis of synaptic nuclear protein spectrum disorders. For effective tracking of the health status of a high-risk population of PD, such as people with a family history of PD, using α-synuclein may be helpful in the early diagnosis of PD and the monitoring of disease progression during treatment, so as to provide a basis for new treatment as well as theoretical guidance for the further study on the pathogenesis of PD.

## 5 Conclusion

Our study supports that there is potential in its use as a biomarker but still there are issues to be overcome. As the work herein is validated in the future, new therapeutic strategies can be directly adopted to prevent and delay the disease progression in PD in the early stages of α-synuclein movement.

## Author contributions

**Conceptualization:** Fudong Yan, Qinyong Ye.

**Data curation:** Fudong Yan, Ying Chen, Min Li, Yingqing Wang, Wenming Zhang, Xiaochun Chen, Qinyong Ye.

**Formal analysis:** Fudong Yan, Ying Chen, Min Li, Yingqing Wang, Wenming Zhang, Xiaochun Chen, Qinyong Ye.

**Funding acquisition:** Qinyong Ye.

**Investigation:** Qinyong Ye.

**Methodology:** Fudong Yan, Ying Chen, Min Li, Yingqing Wang, Wenming Zhang, Xiaochun Chen, Qinyong Ye.

**Project administration:** Fudong Yan, Xiaochun Chen, Qinyong Ye.

**Resources:** Yingqing Wang, Wenming Zhang, Xiaochun Chen, Qinyong Ye.

**Software:** Fudong Yan, Yingqing Wang, Wenming Zhang, Xiaochun Chen, Qinyong Ye.

**Supervision:** Fudong Yan, Xiaochun Chen, Qinyong Ye.

**Validation:** Fudong Yan, Min Li, Yingqing Wang, Wenming Zhang, Xiaochun Chen, Qinyong Ye.

**Visualization:** Fudong Yan, Wenming Zhang, Xiaochun Chen, Qinyong Ye.

**Writing – original draft:** Fudong Yan, Qinyong Ye.

**Writing – review & editing:** Fudong Yan, Qinyong Ye.

## References

[1] Dick FD Parkinson's disease and pesticide exposures. Br Med Bull 2006;79–80:219–31. 10.1093/bmb/ldl018 17242039

[2] Tansey MG Goldberg MS Neuroinflammation in Parkinson's disease: its role in neuronal death and implications for therapeutic intervention. Neurobiol Dis 2010;37:510–8. 19913097 10.1016/j.nbd.2009.11.004 PMC2823829

[3] Gelb DJ Oliver E Gilman S Diagnostic criteria for Parkinson disease. Arch Neurol 1999;56:33–9. 9923759 10.1001/archneur.56.1.33

[4] Dickson DW Braak H Duda JE Neuropathological assessment of Parkinson's disease: refining the diagnostic criteria. Lancet Neurol 2009;8:1150–7. 19909913 10.1016/S1474-4422(09)70238-8

[5] Goedert M Alpha-synuclein and neurodegenerative diseases. Nat Rev Neurosci 2001;2:492–501. 11433374 10.1038/35081564

[6] George JM Jin H Woods WS Characterization of a novel protein regulated during the critical period for song learning in the zebra finch. Neuron 1995;15:361–72. 7646890 10.1016/0896-6273(95)90040-3

[7] Vance JM1 Ali S Bradley WG Gene-environment interactions in Parkinson's disease and other forms of parkinsonism. Neurotoxicology 2010;31:598–602. 20430055 10.1016/j.neuro.2010.04.007

[8] Chartier-Harlin MC Kachergus J Roumier C Alpha-synuclein locus duplication as a cause of familial Parkinson's disease. Lancet 2004;364:1167–9. 15451224 10.1016/S0140-6736(04)17103-1

[9] Mrabet S Ben Ali N Achouri A Gastrointestinal dysfunction and neuropathologic correlations in Parkinson disease. J Clin Gastroenterol 2016;50:e85–90. 27433810 10.1097/MCG.0000000000000606

[10] Stokholm MG Danielsen EH Hamilton-Dutoit SJ Pathological α-synuclein in gastrointestinal tissues from prodromal Parkinson disease patients. Ann Neurol 2016;79:940–9. 27015771 10.1002/ana.24648

[11] Beach TG1 Adler CH Sue LI Multi-organ distribution of phosphorylated alpha-synuclein histopathology in subjects with Lewy body disorders. Acta Neuropathol 2010;119:689–702. 20306269 10.1007/s00401-010-0664-3 PMC2866090

[12] Braak H del Tredici K Rüb U Staging of brain pathology related to sporadic Parkinson's disease. Neurobiol Aging 2003;24:197–211. 12498954 10.1016/s0197-4580(02)00065-9

[13] Gershon MD Erde SM The nervous system of the gut. Gastroenterology 1981;80:1571. 6112192

[14] Gershon MD The enteric nervous system: a second brain. Hosp Pract 1999;34:31–42. 10.3810/hp.1999.07.153 10418549

[15] Heanue TA Pachnis V Enteric nervous system development and Hirschsprung's disease: advances in genetic and stem cell studies. Nat Rev Neurosci 2007;8:466–79. 17514199 10.1038/nrn2137

[16] Minguez-Castellanos A Chamorro CE Escamilla-Sevilla F Do alpha-synuclein aggregates in autonomic plexuses predate Lewy body disorders? A cohort study. Neurology 2007;68:2012–8. 17548551 10.1212/01.wnl.0000264429.59379.d9

[17] Wang J Bi M Liu H The protective effect of lactoferrin on ventral mesencephalon neurons against MPP + is not connected with its iron binding ability. Sci Rep 2015;5:10729. 26035688 10.1038/srep10729 PMC4451802

[18] Holmqvist S Chutna O Bousset L Direct evidence of Parkinson pathology spread from the gastrointestinal tract to the brain in rats. Acta Neuropathol 2014;128:805–20. 25296989 10.1007/s00401-014-1343-6

[19] Svensson E Horvath-Puho E Thomsen RW Vagotomy and subsequent risk of Parkinson's disease. Ann Neurol 2015;78:522–9. 26031848 10.1002/ana.24448

[20] Derkinderen P Rouaud T Lebouvier T Parkinson disease: the enteric nervous system spills its guts. Neurology 2011;77:1761–7. 22067963 10.1212/WNL.0b013e318236ef60

[21] Lebouvier T Neunlist M Bruley des Varannes S Colonic biopsies to assess the neuropathology of Parkinson's disease and its relationship with symptoms. PLoS One 2010;5:e12728. 20856865 10.1371/journal.pone.0012728 PMC2939055

[22] Shannon KM Keshavarzian A Mutlu E Alpha-synuclein in colonic submucosa in early untreated Parkinson's disease. Mov Disord 2012;27:709–15. 21766334 10.1002/mds.23838

[23] Sanchez-Ferro A Rabano A Catalan MJ In vivo gastric detection of alpha-synuclein inclusions in Parkinson's disease. Mov Disord 2015;30:517–24. 25113060 10.1002/mds.25988

[24] Shannon KM Keshavarzian A Dodiya HB Is alpha-synuclein in the colon a biomarker for premotor Parkinson's disease? Evidence from 3 cases. Mov Disord 2012;27:716–9. 22550057 10.1002/mds.25020

[25] Movement Disorders, Parkinson's Disease Group NBoCMA. Diagnosis for Parkinson's Disease. Chin J Neurol 2006;39:408–9.

[26] Jacobs TW Gown AM Yaziji H Comparison of fluorescence in situ hybridization and immunohistochemistry for the evaluation of HER-2/neu in breast cancer. J Clin Oncol 1999;17:1974–82. 10561247 10.1200/JCO.1999.17.7.1974

[27] Parkinson J An essay on the shaking palsy. 1817. J Neuropsychiatry Clin Neurosci 2002;14:223–36. 11983801 10.1176/jnp.14.2.223

[28] Kordower JH Olanow CW Dodiya HB Disease duration and the integrity of the nigrostriatal system in Parkinson's disease. Brain 2013;136:2419–31. 23884810 10.1093/brain/awt192 PMC3722357

[29] Gasser T Genomic and proteomic biomarkers for Parkinson disease. Neurology 2009;72:S27–31. 19221311 10.1212/WNL.0b013e318198e054

[30] Ruffmann C Parkkinen L Gut feelings about β-synuclein in gastrointestinal biopsies: biomarker in the making? Mov Disord 2016;31:193–202. 26799450 10.1002/mds.26480 PMC4755164

[31] Kahle PJ alpha-Synucleinopathy models and human neuropathology: similarities and differences. Acta Neuropathol 2008;115:87–95. 17932682 10.1007/s00401-007-0302-x

[32] Dickson DW Fujishiro H DelleDonne A Evidence that incidental Lewy body disease is pre-symptomatic Parkinson's disease. Acta Neuropathol 2008;115:437–44. 18264713 10.1007/s00401-008-0345-7

[33] Gray MT Munoz DG Gray DA Alpha-synuclein in the appendiceal mucosa of neurologically intact subjects. Mov Disord 2014;29:991–8. 24352892 10.1002/mds.25779

[34] Croisier EDEM Deprez MKG Dexter DTRKBP Comparative study of commercially available anti-alpha-synuclein antibodies. Neuropathol Appl Neurobiol 2006;32:351–6. 16640654 10.1111/j.1365-2990.2006.00722.x

[35] Kovacs GG Wagner U Dumont B An antibody with high reactivity for disease-associated alpha-synuclein reveals extensive brain pathology. Acta Neuropathol 2012;124:37–50. 22370907 10.1007/s00401-012-0964-x

